# Pitfalls in the Serological Diagnosis of Primary Human Cytomegalovirus Infection in Pregnancy Due to Different Kinetics of IgM Clearance and IgG Avidity Index Maturation

**DOI:** 10.3390/diagnostics11030396

**Published:** 2021-02-26

**Authors:** Antonella Sarasini, Alessia Arossa, Maurizio Zavattoni, Chiara Fornara, Daniele Lilleri, Arsenio Spinillo, Fausto Baldanti, Milena Furione

**Affiliations:** 1 Virologia Molecolare, Microbiologia e Virologia, Fondazione IRCCS Policlinico San Matteo, 27100 Pavia, Italy; a.sarasini@smatteo.pv.it (A.S.); m.zavattoni@smatteo.pv.it (M.Z.); c.fornara@smatteo.pv.it (C.F.); d.lilleri@smatteo.pv.it (D.L.); f.baldanti@smatteo.pv.it (F.B.); 2 Ostetricia e Ginecologia, Fondazione IRCCS Policlinico San Matteo, 27100 Pavia, Italy; a.arossa@smatteo.pv.it (A.A.); a.spinillo@smatteo.pv.it (A.S.); 3 Dipartimento di Scienze Clinico-Chirurgiche, Diagnostiche e Pediatriche, Università di Pavia, 27100 Pavia, Italy

**Keywords:** HCMV primary infection, pregnancy, IgM antibody, IgG antibody, IgG avidity, DNAemia, dating of infection onset

## Abstract

Primary infection occurs when seronegative women are infected by human cytomegalovirus (HCMV). Diagnosis of primary infection is based on the following: antibody seroconversion, presence of IgM and low IgG avidity index (AI), and presence of DNAemia. The kinetics of HCMV-specific IgM antibody and maturation of AI might be very rapid or long-lasting during primary infection, which makes serological diagnosis insidious. The aims of this study were as follows: (i) to report atypical kinetics of HCMV-specific IgM antibody and AI early after onset of primary HCMV infection in a population of pregnant women, and (ii) to assess the frequency of such results. Altogether, 1309 sequential serum samples collected from 465 pregnant women with primary HCMV infection were included in the study. As a general rule, using the LIAISON^®^CMVIgMII and LIAISON^®^CMVIgGAvidityII assays, virus-specific IgM antibody levels decreased, while IgG AI increased over time during the first three months after infection onset. However, early clearance of IgM antibody and/or early IgG AI maturation occurred in 46/426 (10.7%) women. In more details, 20/426 (4.7%) and 26/418 (6.2%) women had undetectable IgM antibody or high IgG AI, respectively, when tested within 1–3 months after well-defined infection onset. Twenty sera from as many women with high IgG AI by the LIAISON assay were further tested for IgG AI by VIDAS^®^CMVIgGAvidityII and Mikrogen *recom*LineCMVIgG Avidity assays. Comparable results were obtained with VIDAS, whereas 14/20 sera gave low AI with the Mikrogen assay. In conclusion, about 11% of pregnant women undergoing a primary HCMV infection showed misleading serological results. Additional and appropriate testing might help in reducing the risk of missing HCMV primary infection in pregnancy. Furthermore, preconceptional testing should be strongly recommended.

## 1. Introduction

Human cytomegalovirus (HCMV) infection is the most common causes of congenital infection, impairing child development worldwide [1], with an overall estimated prevalence of 0.7% [2]. The rate of vertical transmission is about 30–40% in pregnant women with primary infection [2,3], and is believed to be much lower (around 1%) in pregnant women with preconception immunity [2,4]. Moreover, the most severe *sequelae* in congenitally infected newborns are the consequence of a primary infection occurring during the first trimester of pregnancy [5,6,7]. Thus, diagnosis or exclusion of primary infection, as well as definition of the gestational age at which primary maternal infection occurred, represent critical points for a correct clinical management of pregnant women.

According to the literature, primary HCMV infection in pregnancy is ascertained (i) when IgG seroconversion is documented and (ii) in the presence of HCMV-specific IgM antibody and low IgG avidity index (AI). The AI currently includes three levels: low avidity, which is generally associated with a recent primary infection occurring within the last 3 months; high avidity, which excludes the onset of primary infection in the last 3 months; or intermediate avidity, which does not allow reliable discrimination between recent and non-recent primary infection. Recent studies reported a good concordance among commercial assays for IgG AI determination [8,9]. However, in rare cases, specific IgM antibody might be rapidly cleared [10] and a high AI could be detected within 90 days after onset of primary infection [11]. Absence of specific IgM and/or a high AI in sera collected early after the onset of primary infection makes the serological diagnosis of primary infection very difficult.

In our center, for diagnostic purposes, a panel of multiple serological and molecular assays are performed on sequential blood samples for diagnosis and dating of primary HCMV infection in pregnant women. Using this approach, during the last years, a fair number of cases of primary infection with early clearance of specific IgM antibody or early high AI were detected.

The aims of this retrospective study were as follows: (i) to report about atypical kinetics of virus-specific IgM antibody response and early IgG avidity maturation within 90 days after the onset of primary HCMV infection in a population of pregnant women; (ii) to assess the frequency of such results together with the resulting risk of missing or misdiagnosing a primary HCMV infection in pregnancy.

## 2. Materials and Methods

### 2.1. Patients and Samples

This study was restricted to the following: (i) a time period when commercial assays for HCMV IgG, IgM, and AI were available in association with the other serological and molecular assays in use in our center for the diagnosis of primary HCMV infection, and (ii) cases of primary HCMV infection with a well-defined onset of infection. The study population was identified within the group of women who were referred to our institution in Pavia, Italy, for confirmation/interpretation of a HCMV IgM-positive result obtained elsewhere. Virologic results were retrospectively reviewed and kinetics of (i) IgM antibody clearance, (ii) IgG avidity maturation, and (iii) DNAemia over time were re-examined in this population. Time intervals considered were 1–30, 61–90, 91–120, 121–180, and >180 days after onset of primary infection.

### 2.2. Diagnosis and Timing of Primary Maternal HCMV Infection

Diagnosis and dating of primary HCMV infection were achieved prospectively for clinical management based on two or more of the following criteria, as previously reported [12]: (i) appearance of HCMV-related symptoms as well as biochemical and hematological signs associated with HCMV infection; (ii) IgG seroconversion; (iii) seroconversion of neutralizing antibodies (Nt), which occurs 4–6 weeks after onset of primary infection in human fibroblast cell cultures [13]; (iv) kinetics of HCMV-specific IgM and IgG antibodies; (v) low IgG AI; and (vi) presence of HCMV DNA in blood [14]. For diagnostic purposes, HCMV-specific IgG was determined by LIAISON^®^CMVIgGII assay (DiaSorin, Saluggia, Italy), while IgM antibody was determined by LIAISON^®^CMVIgMII assay (chemiluminescent immunoassay (CLIA) IgM) and ENZY-WELL Cytomegalovirus IgM (DIESSE Diagnostica Senese SpA, Monteriggioni, Siena, Italy) (ELISA IgM). IgG AI was determined by LIAISON^®^CMVIgGAvII assay. Retrospectively, when serological data were reviewed, the analysis was restricted to women with well-defined infection onset. Two additional assays were retrospectively performed on sera with a high AI within 90 days after onset of HCMV infection: VIDAS^®^CMVIgGAvidity II (bioMerieux, Marcy-l’Etoile, France) and *recom*LineCMVIgG Avidity (Mikrogen Diagnostik, MIKROGEN GMBH, Neuried, Germany). The *recom*LineCMVIgG Avidity is a line immunoassay based on highly purified recombinant HCMV antigens fixed on nitrocellulose membrane test strips (IE1, CM2, p150, p65, gB1, and gB2). According to the manufacturer’s instructions, low avidity is defined as more than 50% reduction in band intensity in two of the four antigens: IE1, CM2, p150, and gB2.

## 3. Results

### 3.1. Characteristics of the Study Population

To restrict our study to a period when a commercial serological system for HCMV IgG, IgM, and AI determination was available, we referred to the period October 2015–November 2018, when 1523 samples from 551 women with primary HCMV infection were prospectively collected and tested. The onset of primary infection was presumed by considering clinical, biochemical/hematological, serological, and molecular parameters when the women were admitted to our centre. Of the 551 women, 48 were not pregnant, 38 had a pre-conceptional infection, and 465 had a primary infection occurring during pregnancy. In detail, primary infection occurred periconceptionally (i.e., within 4 weeks after the last menstrual period (±2 weeks after or before the presumed time of conception [15])) in 135/465 (29%) women, while it occurred during the first, second, and third trimester in 153/465 (32.9%), 134/465 (28.9%), and 43/465 (9.2%) women, respectively. Overall, the results of 1309 sequential samples from the 465 women with primary infection during pregnancy were considered for this study to evaluate the kinetics of the following: (i) specific HCMV CLIA and ELISA IgM antibody, (ii) IgG AI maturation, and (iii) HCMV DNAemia.

The first available sample was collected after a median time of 36 (range 3–120) days after the estimated onset of infection and the subsequent sequential samples (median 2; range 1–9 per patient) were obtained monthly for a median follow-up period of 92 (range: 28–570) days after onset of infection.

### 3.2. Kinetics of HCMV CLIA IgM Antibody

**Figure 1:**
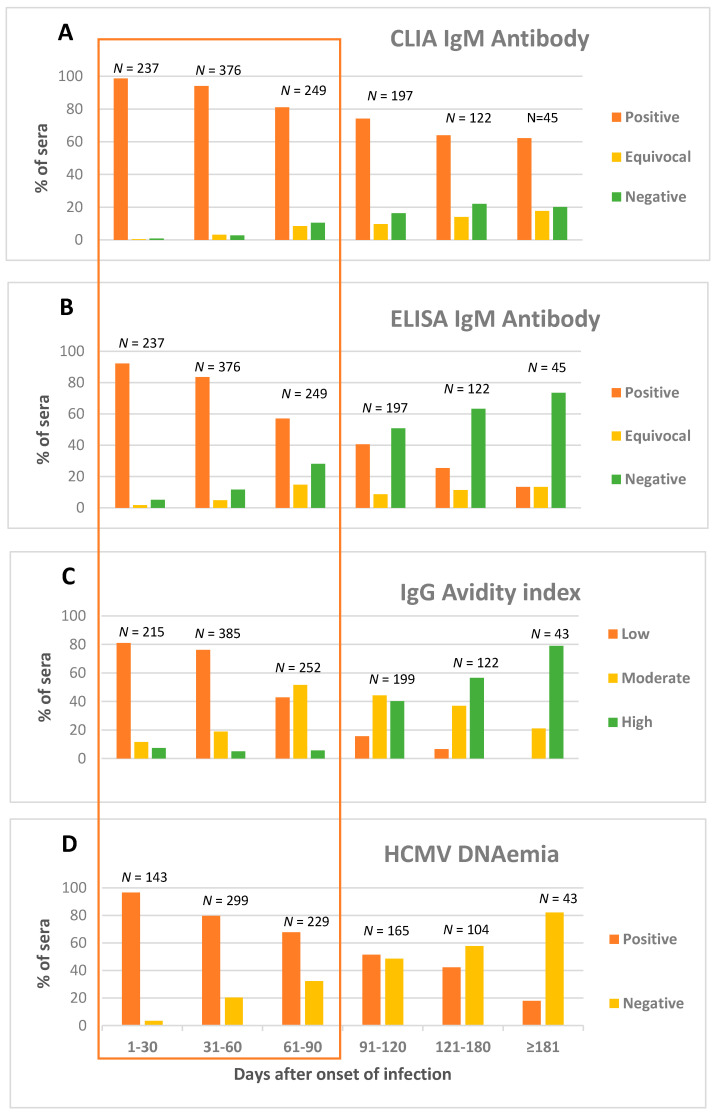
Human cytomegalovirus (HCMV) chemiluminescent immunoassay (CLIA) IgM (**A**) and enzyme-linked immunosorbent assay (ELISA) IgM (**B**) antibody, IgG avidity index (**C**), and DNAemia (**D**) results, as obtained on sequential serum and whole blood samples from 465 pregnant women with well-defined primary HCMV infection.

At a preliminary analysis, 34 sera collected from 27 women within 90 days after infection onset (ten sera in the interval 31–60, and twenty-four in the interval 61–90 days), were HCMV CLIA IgM-negative, as shown in Figure 1A. To be more rigorous, the diagnostic criteria used to define the onset of infection were reviewed in order to restrict the following analysis to women with well-defined infection onset. Because the diagnostic criteria to date the onset of infection were weak, seven women (9 sera) were excluded from further analysis. Among the remaining 20 women, 16 showed IgG (*n* = 8), Nt (*n* = 5), or IgG + Nt (*n* = 3) seroconversion. Among them, all showed a low AI and 15/16 presence of DNAemia. The four women who did not show IgG or Nt seroconversion showed signs/symptoms of infection, increase in IgG levels, low AI, and presence of DNAemia, and three of them were seronegative before pregnancy. Overall, if considering only these 20 cases of infection with a well-defined onset, HCMV CLIA IgM was negative in 2.9% (25/862) of sera from 4.7% (20/426) of women. The virological follow-up of two patients with low levels of CLIA IgM during the early phase of infection is shown in Table 1 (pt#1 and #2).

Conversely, even though IgM antibodies usually decrease over time during most HCMV primary infections, more than 60% (28/45) of sera tested ≥181 days after onset of primary infection were still CLIA IgM-positive.

Taken together, these findings indicate that, considering the kinetics of HCMV-specific IgM CLIA antibody, there are three groups of patients: (i) about 5% of women showing early clearance (within 3 months after onset of infection), (ii) about 35% of them testing IgM-positive for 6 months, and (iii) about 60% having a long-lasting IgM persistence (>6 months).

### 3.3. Kinetics of HCMV ELISA IgM Antibody

During the first 90 days after onset of primary infection, 14.6% (126/862) of sera from 20.9% (89/426) of pregnant women were already negative for HCMV-specific ELISA IgM. In particular, as shown in Figure 1B, absence of ELISA IgM antibody was observed in 5.1% (12/237), 11.7% (44/376), and 28.1% (70/249) of sera collected during the first, second, and third month after the onset of primary infection, respectively. Conversely, only 13.3% (6/45) of sera tested were still ELISA IgM-positive ≥181 days after onset of primary infection.

These findings suggest that the format of the serological assay (ELISA vs. CLIA) is crucial to detect HCMV-specific IgM antibody, with the CLIA being more sensitive and the ELISA being more specific.

### 3.4. Kinetics of HCMV IgG Avidity

At a preliminary analysis, 74 sera (17, 23, and 34 in the time interval 1–30, 31–60, and 61–90 days after onset, respectively) from 48 women showed a high AI within 90 days after onset of infection. As for IgM kinetics, the diagnostic criteria used to define the onset of infection for these women were reviewed, in order to restrict the analysis to cases with a well-defined onset of infection. Among the 48 women, 22 were excluded from further analysis because they did not satisfy the criteria for dating onset of primary infection. When considering only the cases with a well-defined onset of infection, a high AI was observed in 5.8% (49/852) of sera collected <90 days after infection onset from 26/418 women (6.2%). In particular, as shown in Figure 1C, high AI was observed in 7.4% (16/215), 5% (19/385), and 5.6% (14/252) of samples collected during the first, second, and third month after the onset of primary infection, respectively. The virological follow-up of two patients with high AI during the early phase after infection onset is shown in Table 1 (pt#3 and #4).

Serum samples with high AI collected from 20/26 women within 90 days after onset of infection were available for retrospective testing with another avidity assay. The VIDAS^®^CMVIgGAvidity II showed good concordance with the LIAISON assay. In particular, 15/20 samples showed high AI, whereas 5 and 1 showed moderate and low AI, respectively. On the other hand, using *recom*LineCMVIgG Avidity, 14/20 samples showed results suggestive of a recent HCMV infection that occurred within the previous 6–8 weeks (*n* = 4) or 14 weeks (*n* = 10). These findings suggest that different AI results can be obtained using assays based on different formats, such as CLIA versus immunoblotting.

Even though LIAISON AI usually increases over time during primary infection, 15.6% (31/199) and 6.6% (8/122) of sera collected in the time interval 91–120 and 121–180 days after infection, respectively, showed a low AI (Figure 1C). In these cases, a recent primary infection should be suspected even though the onset of primary HCMV infection occurred more than 3 months in advance.

### 3.5. HCMV DNAemia

Presence of HCMV DNAemia was observed in 79.1% (531/671) of blood samples collected within the first 90 days after infection onset from 346/457 (75.7%) pregnant women (Figure 1D). On the other hand, 18% (7/39) of the blood samples tested were still HCMV DNA-positive ten months after onset of infection. These findings suggest that, in the presence of HCMV DNAemia, a primary infection should be consistently taken into consideration.

## 4. Discussion

According to the literature [10,16], diagnosis of primary infection in pregnancy is based on the following: (i) IgG seroconversion on sequential serum samples, and/or (ii) presence of IgG and IgM antibody, and low AI.

In our study, during the first three months after onset of primary infection, about 5% of women were CLIA IgM-seronegative. This finding seems to be due to the serological assay used, because absence of IgM in the same time interval was observed more frequently (20%) when an ELISA IgM assay was used. Although IgM response may be very transient and go undetected even in a recent primary infection, to the best of our knowledge, this is the first time that the early clearance of IgM antibody during the first three months after onset of primary HCMV infection has been described in a population of pregnant women, in whom stringent criteria for diagnosis and dating of primary infection were used. In particular, among the population of pregnant women described, besides a group who tested IgM-positive for about 6 months, two additional groups of patients were observed: (i) those who showed an early clearance of specific IgM, within 3 months after onset of infection; and (ii) those who had a long-lasting persistence, more than 6 months after infection onset. According to the manufacturer’s instructions, AI should be determined only in IgM-positive sera. It was previously suggested that this approach could be unsatisfactory, because a few cases of primary HCMV infection could be missed [11]. Our study confirms this result and shows that, by using this algorithm, diagnosis of a recent primary infection may be missed in about 5% of women.

The avidity test is a very important tool for the diagnosis of primary infection because AI mostly increases over time, being low during the first three months after onset of infection, and then increasing to higher levels. Nevertheless, differences in the kinetics of IgG avidity maturation were recently reported [17,18,19] in pregnant women with primary infection. In this study, about 6% of women with sera collected in the first three months after the onset of primary infection showed a high AI. Most (15/20) LIAISON-high AI results were confirmed using the VIDAS^®^CMVIgGAvidity II, but discordant results were observed using *recom*LineCMVIgG Avidity. In particular, 14/20 sera showed a recent infection by the latter assay. Recent studies reported a good concordance among different commercial avidity assays [8,9], but our findings suggest that different results could be obtained by using different AI assays, and further studies should be done to clarify this point.

From this study, it can be concluded that both rapid clearance of specific IgM antibody and fast AI maturation lead to misdiagnosis of 10.7% (46/426) of recent HCMV primary infections in pregnant women, with consequences for the obstetrical/neonatal clinical management of these pregnancies. Furthermore, in these cases, as the risk of vertical transmission after primary infection is about 30%, a congenital infection could be erroneously attributed to a maternal non-primary HCMV infection, as recently reported in a population of French women [20]. In particular, according to this study, a primary infection was excluded when (i) absence of IgM or (ii) presence of IgM and high AI were observed during the first 12–16 week of pregnancy. In addition, another study considered the presence of IgM, high AI, and HCMV DNA in blood or in other bodily fluids within 16 gestation weeks as diagnostic for non-primary infection during pregnancy [21].

Persistence of IgM-positivity for many weeks after onset of primary infection is well-known. In our study, this depended on the format of the IgM method used: in particular, 62% (28/45) of women were still CLIA IgM-positive beyond 180 days after onset of infection, whereas only 13% (6/45) were ELISA IgM-positive. On the other hand, about 12% of women (39/321) showed slow kinetics of avidity maturation, with a low AI in the time interval 91–180 days after onset of infection. Both these events, persistence of IgM-positivity and slow AI maturation in pregnant women, can lead to an erroneous diagnosis of recent primary infection during pregnancy with a subsequent incorrect management of pregnancy.

HCMV DNAemia was positive in about 80% of samples collected <90 days after infection. DNAemia-positivity mostly decreases over time, as our group had previously reported [12]. In our experience, this test is very useful, because, in the case of the presence of DNAemia, a recent primary infection should always be suspected and investigated.

This study underlines the fact that the interpretation of HCMV serological results during pregnancy can be very challenging. To reduce the possibility of missing the diagnosis of primary HCMV infection during pregnancy, the use of a panel of multiple serological and molecular assays, associated with a careful anamnesis and evaluation of clinical and biochemical parameters, should be strongly recommended. In particular, the serological panel could consist of the following: (i) two IgM antibody assay based on different methods, i.e., CLIA, ELISA, or immunoblotting; (ii) two AI assays based on different methods, i.e., CLIA and immunoblotting; and (iii) evaluation of Nt antibodies in human fibroblast cell cultures.

Finally, a preventive approach done by testing women of childbearing age before conception can provide additional help, as documented by the following considerations: (i) firstly, knowledge of HCMV serostatus before conception permits counseling of seronegative women about hygienic measures to be taken to reduce the risk of infection [22,23,24,25], while subsequent follow-up during pregnancy would permit the detection of a possible seroconversion, thus reducing the risk of misdiagnosis; (ii) secondly, IgG-seropositive women can be informed that the risk of delivering a symptomatic baby is relatively low, and they do not need further sequential HCMV testing during pregnancy [2,4]; (iii) thirdly, in the case of primary HCMV infection, conception can be postponed for 24–48 weeks, as previously suggested [12] and recently confirmed (National CMV Foundation, Tampa, FL, USA, www.nationalcmv.org, accessed on 25 February 2021).

## Figures and Tables

**Table 1:** Virological results in four representative women with early clearance of IgM antibody (Pt#1 and #2) and early high avidity index (AI) (Pt#3 and #4).

Pt#	Days after Onset of Infection	IgG (CLIA) * U/mL	IgM (CLIA) ° U/mL	IgM ELISA	Avidity Index	NT Titre	DNAemia ^§^ copies/mL
1	12	<12	28.8	Pos	nd	<1:5	84
	23	18.3	37.8	Pos	Low	<1:5	1020
	51	54.9	<18	Neg	Low	1:5	108
	81	66.5	<18	Neg	Mod	1:10	30
2	19	84.9	37.6	Pos	Low	<1:5	nd
	30	86	26.3	Pos	Low	<1:5	nd
	59	81.1	<18	Neg	Low	1:20	120
	88	79.2	<18	Neg	Low	1:20	30
3	−50	<12	<12	Neg	nd	<1:5	nd
	21	50	65.9	Pos	High	<1:5	nd
	56	78.8	30.2	Pos	High	1:10	nd
	63	80.9	27.7	Pos	High	1:10	180
	96	77.4	21.3	Neg	High	1:10	90
4	16	41.7	112	Pos	High	<1:5	nd
	23	54.2	102	Pos	High	1:10	810
	58	73.3	55.5	Pos	High	1:20	30
	77	89.3	46.8	Neg	High	1:20	30
	113	78.3	39.5	Neg	High	1:20	30

Pt#, patient number; *, range: neg < 12, pos > 14; °, range: negative < 18, positive > 22; NT, neutralizing antibody in human fibroblasts, negative <1:5; CLIA, chemiluminescent immunoassay; ELISA, enzyme-linked immunosorbent assay; ^§^ negative < 30; nd, not done.

## Data Availability

Not applicable.

## Abbreviations

AI	avidity index
HCMV	human cytomegalovirus
CLIA	chemiluminescent immunoassay
ELISA	enzyme-linked immunosorbent assay
Nt	neutralizing antibodies
